# Fully-automated production of [^68^Ga]Ga-FAPI-46 for clinical application

**DOI:** 10.1186/s41181-020-00112-x

**Published:** 2020-12-17

**Authors:** Sarah Spreckelmeyer, Matthias Balzer, Simon Poetzsch, Winfried Brenner

**Affiliations:** Charité - Universitätsmedizin Berlin, corporate member of Freie Universität Berlin, Humboldt-Universität zu Berlin, and Berlin Institute of Health, Department of Nuclear Medicine, Augustenburger Platz 1, 13353 Berlin, Germany

**Keywords:** Automated production, Imaging, FAP, FAPI-46, Fibroblast

## Abstract

**Background:**

[^68^Ga]Ga-FAPI-46 is a promising radiopharmaceutical for in vivo detection of the fibroblast activation protein by positron emission tomography. Until now, the synthesis of [^68^Ga]Ga-FAPI-46 has been only performed manually. Our aim was to evaluate the automated synthesis of this radiopharmaceutical on two different commercially available synthesis modules in order to make the tracer readily available for clinical application.

**Results:**

The synthesis of [^68^Ga]Ga-FAPI-46 with different amounts of precursor (10–50 μg) on the Modular Lab PharmTracer (MLPT) and Modular Lab eazy (ML eazy) from Eckert & Ziegler with a customized synthesis template and a customized single-use cassette yielded best results with 50 μg FAPI-46 for clinical multi-dose application. All relevant quality control parameters tested (e.g. sterility, stability and radiochemical purity) were in accordance with the European Pharmacopoeia.

**Conclusions:**

[^68^Ga]Ga-FAPI-46 was successfully synthesized fully-automated on the synthesis modules Modular Lab PharmTracer and ML eazy and is, thus, available for multi-dose application in clinical settings.

## Background

**Fig. 1:**
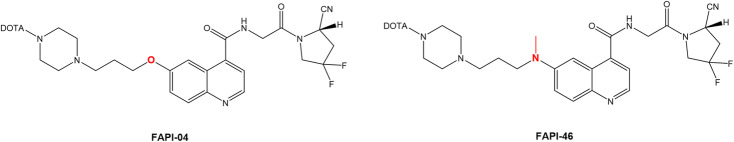
Structure of FAPI-04 (molecular weight: 872.9288 g/mol) and FAPI-46 (molecular weight: 885.96 g/mol)

The fibroblast activation protein (FAP) is an endopeptidase and is located in the cell membrane. FAP is not expressed in normal, healthy tissue, but during wound repair and in the microenvironment of numerous cancer types, including colorectal and pancreatic cancer (Brennen et al. 2012). It has been shown, that FAP is selectively targeted by fibroblast activation protein inhibitors (FAPIs) with a *N*-(4-quinolinoyl)glycyl-(2-cyanopyrrolidine) scaffold (Jansen et al. 2013). In 2018, Lindner et al. developed a series of quinolone-based molecules (e.g. FAPI-04, Fig. 1) for FAP-based imaging and therapy with gallium-68 and lutetium-177, respectively (Lindner et al. 2018). One year later, the same group optimized the structure activity relationships (SARs) and succeeded to improve FAP binding and pharmacokinetics yielding high-contrast images. The most promising molecule from that study is FAPI-46 (Fig. 1) (Loktev et al. 2019). Recently, Giesel et al. reported of FAPI-74, which main advantage is that it can be label with gallium-68 and fluor-18, resulting in [^68^Ga]Ga-FAPI-74 and [^18^F]F-FAPI-74, respectively (Giesel et al. 2020).

To our knowledge, the synthesis of [^68^Ga]Ga-FAPI-46 has not yet been described on an automated synthesis module. Here, we would like to introduce the synthesis conditions of [^68^Ga]Ga-FAPI-46 on two different synthesis modules, namely Modular Lab PharmTracer (MLPT) and Modular Lab eazy (ML eazy) from Eckert & Ziegler Eurotope GmbH – in order to introduce a fully automated [^68^Ga]Ga-FAPI-46 synthesis for clinical application. For that purpose, different loads of FAPI-46 were evaluated based on quality control parameters and the radioactivity distribution on the different cassette parts after synthesis. For the synthesis of ^68^Ga-tracers, different modules are available on the market, which have been reviewed by Boschi et al. (Boschi et al. 2013) In addition, generator post-processing steps like fractionation, anionic-exchange and cation-exchange are described (Boschi et al. 2013). The now proposed fully automated pre-purification and concentration of gallium-68 on a strong cation exchange (SCX) cartridge has the advantage that gallium-68 becomes concentrated while germanium-68 as well as non-radioactive impurities (e.g. Zn^2+)^ are trapped on the SCX cartridge (Velikyan 2015).

## Results

### Labeling results of different FAPI-46 loads on MLPT

The fully automated production of [^68^Ga]Ga-FAPI-46 was conducted on the commercial labeling synthesis modules MLPT and ML eazy. Five different precursor amounts of FAPI-46 were investigated for radiolabeling with gallium-68 (10–50 μg, *n* = 3) on MLPT. Immediately after synthesis, the different cassette parts were analyzed for radioactivity in order to get an overview of the radioactivity distribution on the cassette and reconcile these findings with the effects of the labeling conditions that were used. As shown in Table 1, 30 μg, 40 μg and 50 μg of FAPI-46 resulted in a similar radioactivity distribution in the product vial – 95.1 ± 0.4%, 95.3 ± 1.2% and 96.0 ± 0.6%, respectively. On the CM cartridge, the radioactivity decreases from 2.9 ± 0.3% to 2.1 ± 0.8%. By lowering the amount of FAPI-46 to 10 μg, the percentage found in the product decreased significantly to 70.2 ± 6.1%, and the radioactivity on the CM cartridge increased to 26.2 ± 6.1%. The radioactivity found in the reaction vial, in the waste fraction and on the SCX cartridge however did not change significantly by changing the amount of the starting material.

**Table 1:** Activity measured on different parts of the cassette immediately after synthesis on MLPT (*n* = 3)

load [μg]	product vial [%]	CM cartridge [%]	waste [%]	SCX cartridge [%]	reaction vial [%]	RCY [%]
10	70.2 ± 6.1	26.2 ± 6.1	1.5 ± 1.0	0.6 ± 0.1	1.5 ± 0.3	68.9 ± 3.8
20	91.5 ± 1.9	4.8 ± 0.8	2.1 ± 1.5	0.6 ± 0.3	1.0 ± 0.3	91.8 ± 3.9
30	95.1 ± 0.4	2.9 ± 0.3	0.4 ± 0.2	0.4 ± 0.1	1.1 ± 0.4	94.2 ± 0.8
40	95.3 ± 1.2	2.7 ± 0.9	0.6 ± 0.1	0.3 ± 0.0	1.1 ± 0.3	95.1 ± 2.1
50	96.0 ± 0.6	2.1 ± 0.8	0.5 ± 0.1	0.5 ± 0.2	0.9 ± 0.2	95.2 ± 1.4

### Labeling results of different FAPI-46 loads on ML eazy

On the ML eazy module, we performed experiments with 10–50 μg of FAPI-46 (*n* = 3) at 98 °C reaction temperature (Table 2). As a result, 20–50 μg FAPI-46 load show the highest radioactivity distribution in the product vial. Decreasing the FAPI-46 load to 10 μg leads to a significant decrease in radioactivity measured in the product vial.

**Table 2:** Activity measured on different parts of the cassette immediately after synthesis on ML eazy at 98 °C (*n* = 3)

load [μg]	product vial [%]	CM cartridge [%]	waste [%]	SCX cartridge [%]	reaction vial [%]
10	36.1 ± 15.5	59.7 ± 15.6	0.0 ± 0.0	0.4 ± 0.0	3.7 ± 0.2
20	75.3 ± 18.2	19.3 ± 14.2	0.2 ± 0.2	0.4 ± 0.1	4.8 ± 4.2
30	88.7 ± 10.1	9.2 ± 9.7	0.0 ± 0.0	0.5 ± 0.1	1.6 ± 0.5
40	85.5 ± 11.9	11.0 ± 9.6	0.1 ± 0.1	0.5 ± 0.2	2.9 ± 3.0
50	89.7 ± 6.7	6.1 ± 5.5	0.0 ± 0.0	0.5 ± 0.1	3.7 ± 1.4

**Fig. 2:**
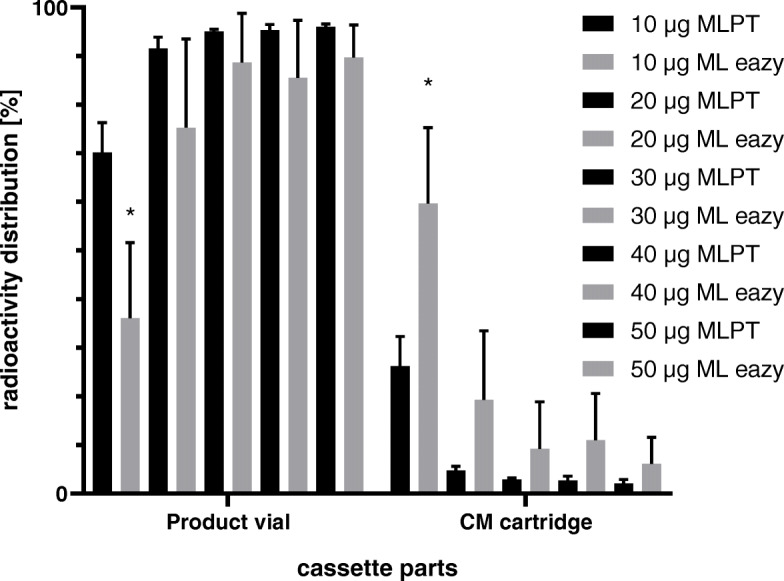
Radioactivity distribution on cassette parts after synthesis on MLPT (95 °C) and ML eazy (98 °C) (dark: MLPT; light: ML eazy) with increasing peptide load from left to right (10 μg–50 μg); * = *p*-value < 0.05

Comparing the distribution pattern of the MLPT with ML eazy (Fig. 2), the results are similar with 20–50 μg FAPI-46 load. Whereas with 10 μg, a significant difference can be observed (70.2% ± 6.1% on MLPT vs. 36.1% ± 15.5% on ML eazy).

### Quality control of [^68^Ga]Ga-FAPI-46

The radiochemical purity was evaluated by *radio*-HPLC and *radio*-iTLC.

**Fig. 3:**
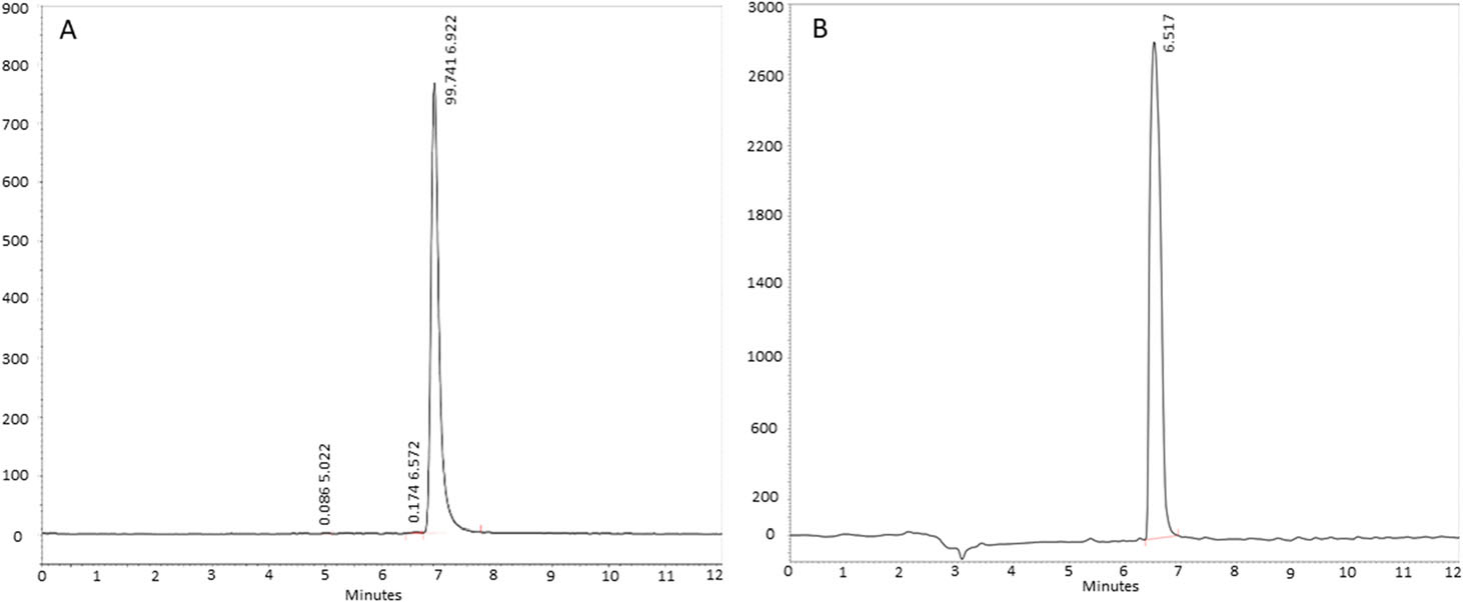
**a** Radio-HPLC Chromatogram of [^68^Ga]Ga-FAPI-46 **b** UV-HPLC Chromatogram of [^nat^Ga]Ga-FAPI-46

With *radio*-HPLC, free gallium-68 was detected at t_R_ = 3.0 min, whereas gallium-68 bound to FAPI-46 was detected at t_R_ = 6.9 min with a purity of 99.7%. Radioactive impurities were detected at t_R_ = 5.0 min with approx. 0.1% and t_R_ = 6.6 min with approx. 0.2% of the total radioactivity. (Fig. 3a).

The cold standard [^nat^Ga]Ga-FAPI-46 shows a similar retention time of t_R_ = 6.5 min as seen in Fig. 3b.

**Fig. 4:**
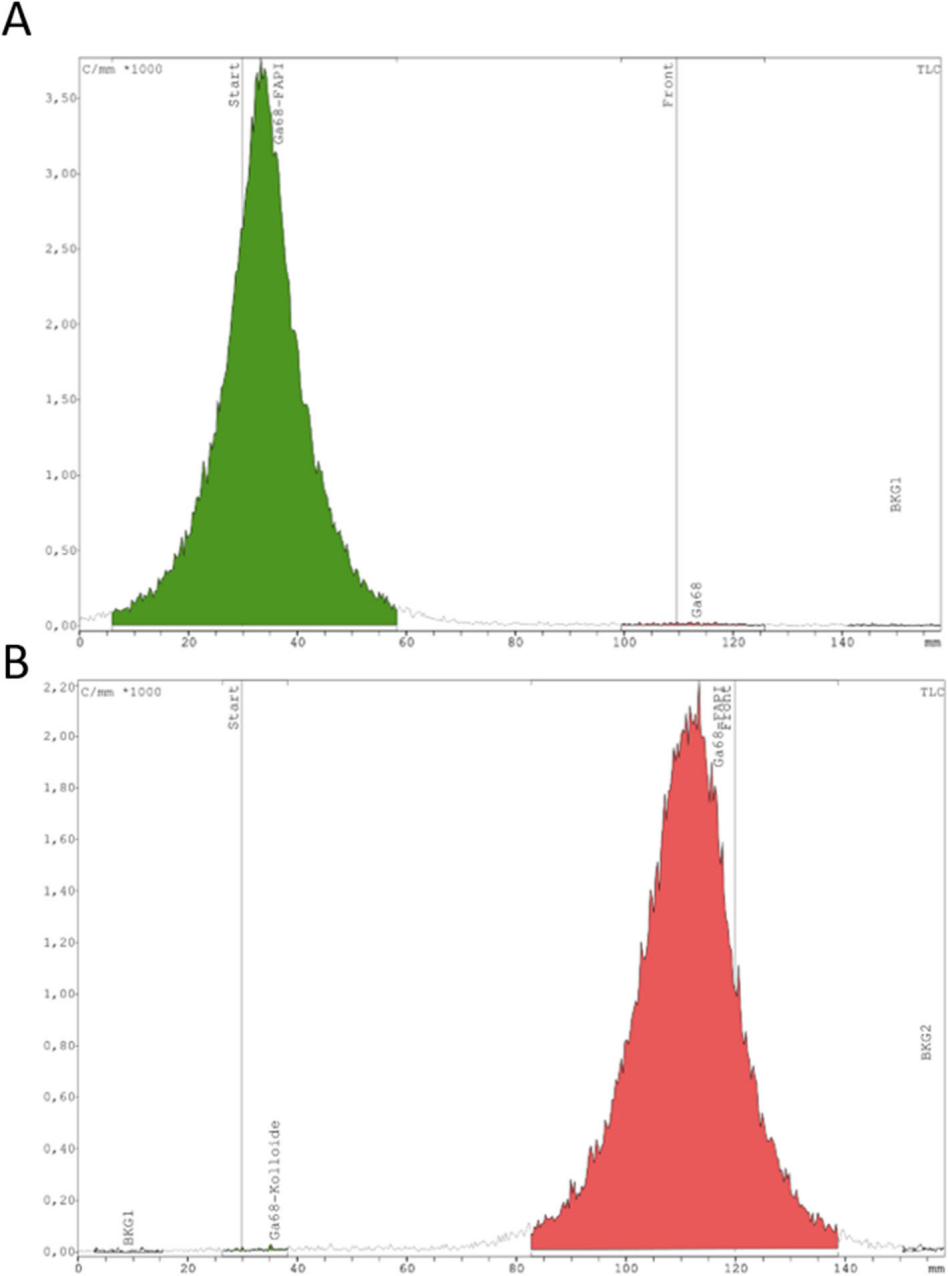
Radio- iTLC- Chromatogram of [^68^Ga]Ga-FAPI-46 (A: Citrate buffer; B: Ammonium acetate/methanol)

With *radio*-iTLC, no free gallium-68 (Fig. 4a) or ^68^Ga-colloide (Fig. 4b) could be detected at R_f_ = 0.8 or R_f_ = 0.2, respectively. The product was detected at R_f_ = 0.2 (Fig. 4a) and R_f_ = 0.8 (Fig. 4b).

In addition, the product solution was tested for endotoxins. For this purpose, the solution was diluted with endotoxin-free-water in a ratio 1:100. For all samples, the endotoxin concentration was below 5.0 IE/mL. This is in accordance with the European Pharmacopoeia (9.0/0125), where a threshold of 175 IE/mL is given. With regard to sterility, all products were sterile.

**Fig. 5:**
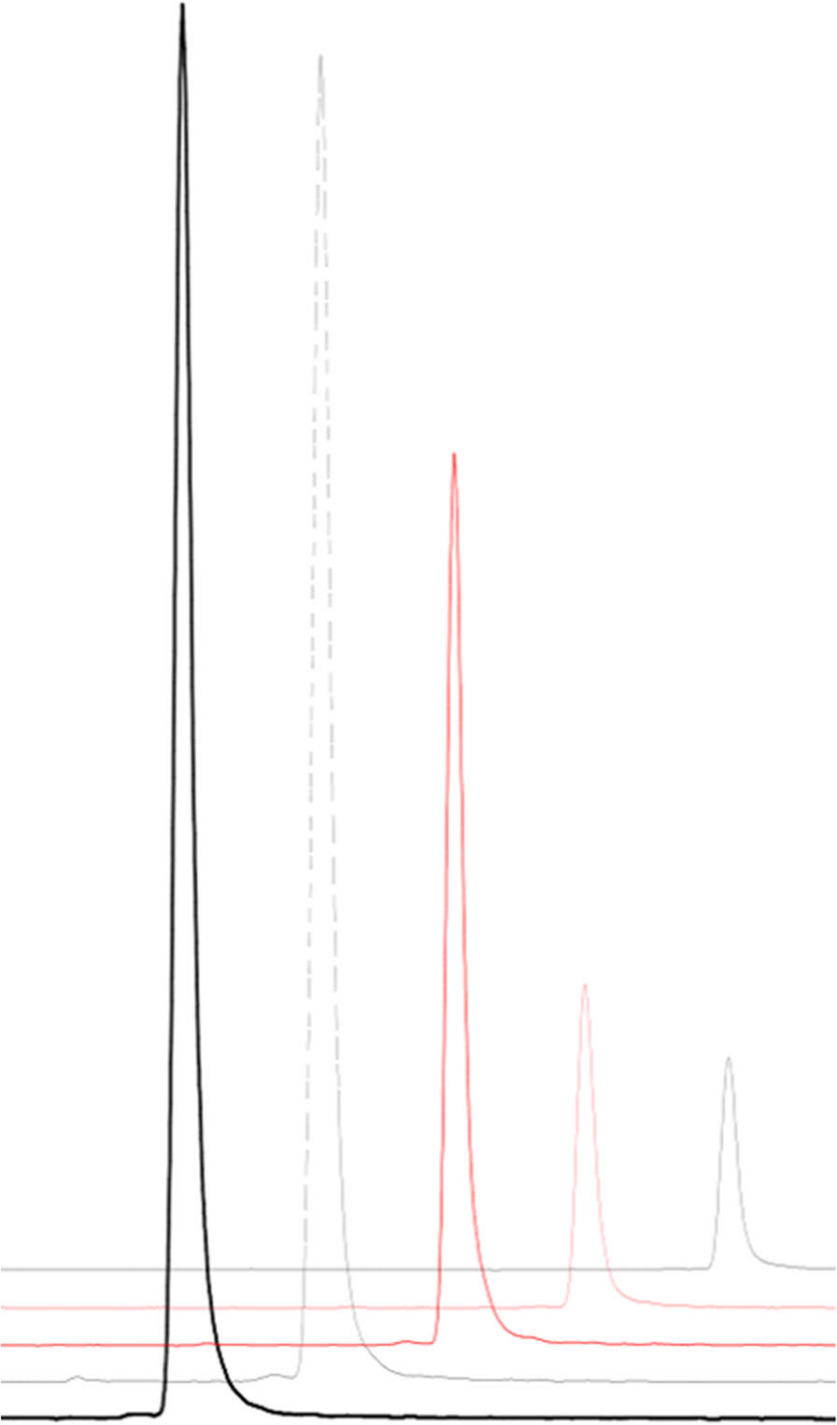
Stability of [^68^Ga]Ga-FAPI-46 (black solid: 0 min, black dash: 30 min, red solid: 1 h, red dash: 2 h, black dot: 3 h)

The stability of [^68^Ga]Ga-FAPI-46 in aqueous solution at room temperature was tested up to 3 h by *radio*-HPLC. As seen in Fig. 5, [^68^Ga]Ga-FAPI-46 is stable under those conditions. No additional radioactive by-products or free gallium-68 could be detected during this time period.

The product specifications of 50 μg FAPI-46 are summarized in Table 3. Acceptance criteria are based on the European Pharmacopoeia (9.0/0125).

**Table 3:** Summary of the product specifications for 50 μg FAPI-46, *n* = 3

Test	Acceptance criteria	[^**68**^Ga]Ga-FAPI-46
Radiochemical purity (*radio*-HPLC)	> 95%	99.7%
Radiochemical purity (*radio*-iTLC)	> 95%	99.9%
pH	4–8.5	4.2
Radioactivity concentration	> 50 MBq/ mL	105–242 MBq/ mL
Radioactivity	> 150 MBq	740–1700 MBq (depending on amount of generators used)
Volume	2–10 mL	7 mL
Color	Colorless	Colorless
Molar radioactivity	1–60 MBq/ nmol	13–30 MBq/nmol
Radionuclidic purity	> 99.9%	99.9%
^68^Ge breakthrough	< 0.001%	0.0002%
Endotoxins	< 19.0 IE/mL	< 5.0 IE/mL
Stability over 3 h	> 90%	99.9%

## Discussion

For establishing the automated synthesis of [^68^Ga]Ga-FAPI-46 on the Modular Lab PharmTracer and ML eazy from Eckert & Ziegler Eurotope GmbH, five different amounts of FAPI-46 were evaluated for the production of this tracer. The results summarized in Table 1 demonstrate that an amount of 30–50 μg of FAPI-46 yield a similar radioactivity distribution on the cassette on MLPT, with 50 μg yielding the lowest amount of uncomplexed gallium-68 on the CM cartridge. Thus, high quantitative radiolabeling was achieved best with 50 μg of FAPI-46. With regard to clinical application from an economical point of view, the application of only 40 μg or even 30 μg FAPI-46 on MLPT looks promising and needs further evaluation by quantitative imaging data. The low standard deviations for all the productions confirm their high reproducibility.

For 10 μg and 20 μg, an increasing amount of radioactivity on the CM cartridge and a decreasing yield of activity in the product vial was observed. Theoretically, two scenarios are possible to our knowledge for explaining the increased radioactivity on the CM cartridge. Either the product is trapped on the CM cartridge, or uncomplexed gallium-68. The product would be eluted from the CM cartridge with 0.9% NaCl solution. However, when eluting the CM cartridge manually with 5 mL of 0.9% NaCl solution after the synthesis, this resulted in no change of the amount of radioactivity measured on the CM cartridge suggesting that no product is retained on the CM cartridge. The most likely explanation therefore is that 10 μg and 20 μg loads are too low to complex ^68^Ga^3+^ ions completely resulting in a trapping of unbound gallium-68 on the CM cartridge.

The minimum amount needed to complex FAPI-46 sufficiently under the described conditions was 30 μg. In former studies in which [^68^Ga]Ga-FAPI-46 was synthesized manually, 20 μg of FAPI-46 were used with a final purification through a solid phase extraction (Oasis light HLB, Waters) and elution with ethanol (Meyer et al. 2019). The radiochemical yields were unfortunately not reported. Thus, a direct comparison to the automated process presented in this manuscript is not possible.

Comparing the radioactivity distribution of MLPT (95 °C) and ML eazy (98 °C), both modules give similar results. For the ML eazy, a higher temperature setting was needed, Since the reaction conditions were the same (e.g. buffer formulation) compared to MLPT, the reason for this finding in our opinion is related to different set-ups of the heating units of ML eazy and MLPT. The heating unit on the ML eazy develops the heat from the bottom, whereas the heating unit on the MLPT provides the heat also from the walls. Thus, we hypothesize, that a higher temperature setting is needed on the ML eazy to reach a homogenous temperature of 95 °C.

With regard to quality control parameters, we achieved an endotoxine-free, sterile and stable solution of [^68^Ga]Ga-FAPI-46 with a radiochemical purity > 95% over a period of 3 h on both MLPT and ML eazy. Moreover, no ethanol had to be used throughout the process, which makes this synthesis favorable over the manual synthesis, because no analysis of solvent residues like ethanol by gas chromatography is necessary. Finally, all tested quality control parameters were in accordance with the European Pharmacopoeia (see Table 3).

## Conclusions

[^68^Ga]Ga-FAPI-46 was successfully synthesized fully-automated on the synthesis modules MLPT and ML eazy for the first time. All the tested quality parameters for radiochemical purity, pH, endotoxins and sterility were in accordance with the European Pharmacopoeia. In addition, the product solution was stable for at least 3 h after production, as shown by *radio*-HPLC. Thus, [^68^Ga]Ga-FAPI-46 can be easily and reliably produced on the modules MLPT and ML eazy for routine clinical application.

## Methods

### Materials

FAPI-46, manufactured by ABX, and the reference standard [^nat^Ga]Ga-FAPI-46 were made available free of costs from SOFIE Biosciences. An aqueous stock solution of 1 mg/mL was prepared and kept at − 15 °C. All chemicals were of pure chemical grade, and solvents for high-pressure-liquid-chromatography (HPLC) were obtained as HPLC grade. TraceSelect water (Sigma-Aldrich) was used in all experiments. The pharmaceutical grade ^68^Ge/^68^Ga generator (GalliaPharm®, Eckert & Ziegler Radiopharma GmbH, Germany), Modular Lab PharmTracer (Eckert & Ziegler Eurotope GmbH, Germany) and reagent set EZ-102 (Eckert & Ziegler Eurotope GmbH, Germany) were used. The amount of detected metal impurities/ ^68^Ge breakthrough as provided by the manufacturer was less than the defined limit in the European Pharmacopeia monograph. Activity counting was performed using a borehole counter (Nuklear-Medizintechnik Dresden GmbH, Germany). HPLC was performed using the HPLC system Knauer Azura (UVD: 2.1 L; P6.1L) coupled with UV and radiometric (Raytest Socket 2″8103 0370) detectors. The TLC scanner MiniGita from Raytest was used. The test for endotoxins was performed using Nexgen PTS (Charles River). The CM cartridge is a cation-exchange cartridge from Sep-Pak Accell Plus light 130 mg sorbent per cartridge, 37–55 μm particle size. The sterility tests were carried out as required by the European Pharmacopoeia by the Institut für Hygiene, Charité.

### Preparation for labeling of FAPI-46 with gallium-68 on MLPT

First, the commercially available fully automated synthesis platform MLPT was equipped with a disposable single-use, customized cassette. The synthesis template and the cassette were altered as follows, as ethanol was excluded from the whole radiolabeling process. The C_18_ cartridge was exchanged by a CM cartridge. The buffer preparations were identical to the ones used for [^68^Ga]Ga-PSMA (sodium ascorbate 0.4 M), with the addition of 300 μg ascorbic acid (0.3 mg in 0.1 mL water). 1.1 mL eluent was used for the extraction of the SCX cartridge. All synthesis reagents were contained in the reagent set except the peptide and ascorbic acid. From the reagent set, 50 mL of 0.9% NaCl was connected to the cassette on the designated spike. FAPI-46 was prepared in 10 μg, 20 μg, 30 μg, 40 μg and 50 μg aliquots from the stock solution (1 mg / mL). Meanwhile, the preparation of the acetate buffer solution was performed according to the user manual using EZ 102. The reaction mixture contains 0.4 mL of the final buffer solution, 0.1 mL of ascorbic acid and an aliquot of FAPI-46, which was loaded into the reaction vial. 3 mL of eluent was added to the eluent vial prior to synthesis.

### Preparation for labeling of FAPI-46 with gallium-68 on ML eazy

First, the commercially available fully automated synthesis platform ML eazy was equipped with a disposable single-use cassette (PSMA cassette). FAPI-46 was prepared in 10–50 μg aliquots from the stock solution (1 mg / mL). The buffer preparations were identical to the ones used for [^68^Ga]Ga-PSMA, with the addition of 300 μg ascorbic acid (0.3 mg in 0.1 mL water). All synthesis reagents were contained in the reagent set except the peptide and ascorbic acid. From the reagent set, 6.0 mL of 0.9% NaCl was filled into the cassette in the designated vial with blue cap. After that, 1.1 mL of the eluent was filled into the designated vial with red cap. The reaction mixture contains 0.4 mL of the final buffer solution, 0.1 mL of ascorbic acid and an aliquot of FAPI-46, which was loaded into the reaction vial (green cap).

### Labeling of FAPI-46 with ^68^GaCl_3_

The synthesis was performed fully-automated without any user interaction. ^68^Ga^3+^ obtained from a 1.850 MBq ^68^Ge/^68^Ga generator (GalliaPharm®) with TiO_2_ matrix, was eluted with 0.1 N HCl. The generator eluate was pre-concentrated on a strong cation exchange (SCX) cartridge. [^68^GaCl_4_]^−^ was recovered from the SCX cartridge by 1.1 mL eluent (5 M NaCl/HCl(0.1 M)) (Schultz et al. 2013). After the ^68^Ga-activity was transferred to the reaction vial, it was heated to 95 °C. After 10 min at 95 °C (or 98 °C on ML eazy), the reaction mixture was cooled down by adding 3.0 mL and 6.0 mL of 0.9% NaCl for MLPT and EAZY, respectively. The crude reaction solution was subsequently transferred through the CM cartridge and a sterile filter into the product vial. The reaction vessel was rinsed with 3.0 mL 0.9% NaCl in case of the MLPT synthesis. A sample for quality control was taken.

### Quality control

After synthesis, the product vial was removed from the MLPT or ML eazy module and further evaluated for quality control determining the following parameters: total product activity, ^68^Ga^3+^-identity via half-life time, chemical purity (pH, sterility and endotoxins) and radiochemical purity (high-pressure-liquid chromatography). The stability of the product at room temperature was monitored by *radio*-HPLC for 3 h. *Radio*-HPLC was performed with the following method. A: acetonitrile + 0.1% TFA; B: water + 0.1% TFA, gradient: 0–8 min A: 0–50%; 8–10 min A: 50%; 10–12 min A: 50–0%. The limit of detection of the radio-HPLC is 10 kBq/ 20 μL injected volume and the recovery is approximately 80%. For iTLC, ammonium acetate/methanol (1/1) and citrate buffer were used as mobile phases and iTLC-SG strips as stationary phase. The sterility tests were carried out as described in the European Pharmacopoeia by the Institut für Hygiene, Charité.

## Data Availability

All data generated or analyzed during this study are included in this published article.

## Abbreviations

FAP: Fibroblast activation protein
FAPI: Fibroblast activation protein inhibitor
n.d.c.: Non decay corrected
RCY: Radiochemical yield
MLPT: Modular Lab PharmTracer
ML eazy: Modular Lab eazy
PET: Positron Emission Tomography
^68^Ga^3 +^: ^68^Gallium^3+^ ion
^68^Ge^4 +^: ^68^Germanium^4+^ ion
HPLC: High-pressure-liquid-chromatography
TFA: Trifluoro acetic acid
